# Allicin, an Emerging Treatment for Pulmonary Arterial Hypertension: An Experimental Study

**DOI:** 10.3390/ijms241612959

**Published:** 2023-08-19

**Authors:** José L. Sánchez-Gloria, Constanza E. Martínez-Olivares, Leonardo Del Valle-Mondragón, Fernando Cortés-Camacho, Oscar R. Zambrano-Vásquez, Rogelio Hernández-Pando, Fausto Sánchez-Muñoz, Laura G. Sánchez-Lozada, Horacio Osorio-Alonso

**Affiliations:** 1 Department of Internal Medicine, Division of Nephrology, Rush University Medical Center, Chicago, IL 60612, USA; jose_sanchez@rush.edu; 2 Experimental Pathology Department, Experimental Pathology Laboratory, Instituto Nacional de Ciencia Médicas y Nutrición “Salvador Zubirán”, Mexico City 14080, Mexico; constanzamtz@hotmail.com (C.E.M.-O.); rogelio.hernandezp@incmnsz.mx (R.H.-P.); 3 Departamento de Farmacología “Dr. Rafael Méndez Martínez”, Instituto Nacional de Cardiología Ignacio Chávez, Mexico City 14080, Mexico; leonardo.delvalle@cardiologia.org.mx; 4 Departamento de Fisiopatología Cardio-Renal, Instituto Nacional de Cardiología Ignacio Chávez, Mexico City 14080, Mexico; fcortesc1700@alumno.ipn.mx (F.C.-C.); renezambrano2513@gmail.com (O.R.Z.-V.); laura.sanchez@cardiologia.org.mx (L.G.S.-L.); 5 Departamento de Inmunología, Instituto Nacional de Cardiología Ignacio Chávez, Mexico City 14080, Mexico; fausto.sanchez@cardiologia.org.mx

**Keywords:** allicin, pulmonary arterial hypertension, endothelial function, renin–angiotensin system, oxidative stress, hypoxia

## Abstract

We assessed whether allicin, through its antihypertensive and antioxidant effects, relieves vascular remodeling, endothelial function, and oxidative stress (OS), thereby improving experimental pulmonary arterial hypertension (PAH). Allicin (16 mg/kg) was administered to rats with PAH (monocrotaline 60 mg/kg). Allicin encouraged body weight gain and survival rate, and medial wall thickness and the right ventricle (RV) hypertrophy were prevented. Also, angiotensin II concentrations in the lung (0.37 ± 0.01 vs. 0.47 ± 0.06 pmoles/mL, allicin and control, respectively) and plasma (0.57 ± 0.05 vs. 0.75 ± 0.064, allicin and control respectively) and the expressions of angiotensin-converting enzyme II and angiotensin II type 1 receptor in lung tissue were maintained at normal control levels with allicin. In PAH rats treated with allicin, nitric oxide (NO) (31.72 ± 1.22 and 51.4 ± 3.45 pmoles/mL), tetrahydrobiopterin (8.43 ± 0.33 and 10.14 ± 0.70 pmoles/mL), cyclic guanosine monophosphate (5.54 ± 0.42 and 5.64 ± 0.73 pmoles/mL), and Ang-(1-7) (0.88 ± 0.23 and 0.83 ± 0.056 pmoles/mL) concentrations increased in lung tissue and plasma, respectively. In contrast, dihydrobiopterin increase was prevented in both lung tissue and plasma (5.75 ± 0.3 and 5.64 ± 0.73 pmoles/mL); meanwhile, phosphodiesterase-5 was maintained at normal levels in lung tissue. OS in PAH was prevented with allicin through the increased expression of Nrf2 in the lung. Allicin prevented the lung response to hypoxia, preventing the overexpression of HIF-1α and VEGF. Allicin attenuated the vascular remodeling and RV hypertrophy in PAH through its effects on NO-dependent vasodilation, modulation of RAS, and amelioration of OS. Also, these effects could be associated with the modulation of HIF-1α and improved lung oxygenation. The global effects of allicin contribute to preventing endothelial dysfunction, remodeling of the pulmonary arteries, and RV hypertrophy, preventing heart failure, thus favoring survival. Although human studies are needed, the data suggest that, alone or in combination therapy, allicin may be an alternative in treating PAH if we consider that, similarly to current treatments, it improves lung vasodilation and increase survival. Allicin may be considered an option when there is a lack of efficacy, and where drug intolerance is observed, to enhance the efficacy of drugs, or when more than one pathogenic mechanism must be addressed.

## 1. Introduction

Pulmonary arterial hypertension (PAH) is a rare subtype of cardiovascular disease [1,2]. PAH is chronic, progressive, and life-threatening, characterized by sustained pulmonary vasoconstriction and irreversible pulmonary vascular remodeling leading to increased pulmonary artery pressure, right heart failure (RHF), and death [3]. The prevalence and incidence of PAH in the population range between 3–5/100,000 and 0.4–0.58/100,000 person-years, respectively [1].

Further, in patients with systemic sclerosis, the PH develops in a low percentage, and endothelial dysfunction, inflammation, fibrosis, severe intrinsic right ventricular (RV) dysfunction, and poor survival in these patients’ features are present [4].

The pathogenic mechanisms involve numerous factors, including bone morphogenetic receptor type 2 (BMPR2) genetic mutations, endothelial dysfunction, oxidative stress (OS), vascular constriction, inflammation, and fibrosis and may even be secondary to other diseases or drug intake [4,5,6,7,8]. More recently, the regulation of gene expression at DNA and mRNA levels has gained interest and has provided relevant information about the pathogenesis of PAH. In this context, studies on DNA methylation, as well as deacetylation, at experimental and clinical levels have provided key details on pathogenic mechanisms involved in the development and progression of PAH, thus opening the possibility of the use of genic therapy as an option in the PAH prophylaxis [7,8,9,10,11]; such mechanisms can be crucial in regulating inflammation-related pathways, pulmonary vascular remodeling, vascular resistance, and RV remodeling [9,10].

On the other hand, in patients with PAH it has been demonstrated that the angiotensin-converting enzyme (ACE)–angiotensin-II (Ang-II)–angiotensin II type 1 receptor (AT1R) axis of the renin–angiotensin system (RAS) is upregulated. Thus, ACE expression is increased in the endothelium of small pulmonary arteries in patients with PAH [12], and the upregulation of AT1R expression in vascular smooth muscle cells (VSMCs) is responsible for the proliferation, migration, and hypertrophy, which leads to medial thickening [13]. Therefore, overactivation of the RAS may cause adverse effects on pulmonary hemodynamics, inducing vasoconstriction [13,14], an effect extensively related to the PAH progression [15]. In addition, it is well known that high levels of Ang-II lead to reactive oxygen species (ROS) production, cellular migration, proliferation, hypertrophy, fibrosis, and inflammation [16]. In this respect, ROS, such as superoxides, are generated by activating the Ang-II-AT1R axis [17]. Furthermore, Ang II increases the hypoxia-induced factor 1 alpha (HIF-1α) expression in VSMCs, causing vascular remodeling [18].

In contrast, the angiotensin-converting enzyme type 2 (ACE2)-Ang-(1-7) axis exhibits vasodilatory effects and can be considered a counterregulatory mechanism of the imbalance between vasoconstrictors and vasodilators caused by RAS activation [15]. Also, the high expression of ang 1-7 has been related to anti-proliferative, anti-fibrotic, and anti-hypertrophic effects in animal models [19,20].

Currently, treatments for PAH are directed toward improve pulmonary vasodilation through nitric oxide (NO)-dependent, prostacyclin, and endothelin pathways [21]. However, the PAH treatments underestimate the pulmonary vascular or RV remodeling, and the natural course of this lethal disease is progression to RV failure and death. Therefore, new treatments for PAH should be aimed at improving pulmonary vasodilation, inhibiting cardiac and vascular remodeling, and improving cardiac function. Allicin, a sulfur compound, has shown beneficial effects through the modulation of RAS components, OS, and improving cardiac function in chronic kidney disease (CKD) [22,23]. Likewise, allicin ameliorates hypertension, improves endothelial function, and decreases right heart hypertrophy [24,25]. A recent study showed that allicin exerts anti-inflammatory and anti-fibrotic effects in experimental PAH, which contributes to preventing pulmonary arterial medial wall thickness [6,25]. Other studies reported that allicin in vivo and in vitro reduced malondialdehyde (MDA) adducts and increased NO release and endothelial nitric oxide synthase (eNOS) expression [21,26], suggesting that allicin can improve endothelial function and therefore vasodilation [6,27,28,29].

Allicin improves endothelial and cardiac function in chronic and cardiovascular diseases, mediated by its anti-inflammatory, antihypertensive, and antioxidant effects. However, the effects of allicin on PAH are not well known and are scarcely studied. Thus, we aimed to assess whether allicin, through its antihypertensive and antioxidant effects, relieves vascular remodeling and improves endothelial function and OS, thereby improving the outcome in the experimental PAH.

## 2. Results

### 2.1. Validation of the PAH Model Using RV Hypertrophy and Lung Vascular Remodeling

**Figure 1:**
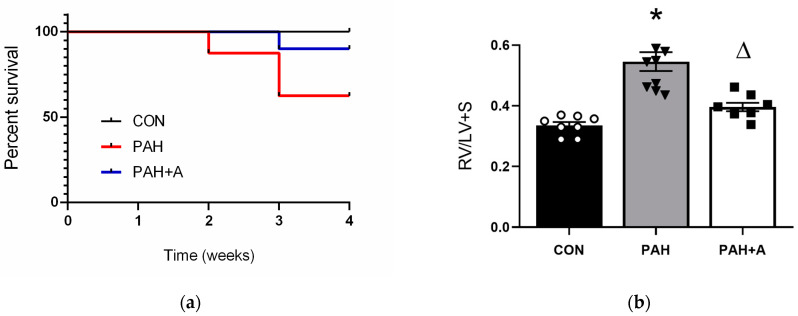

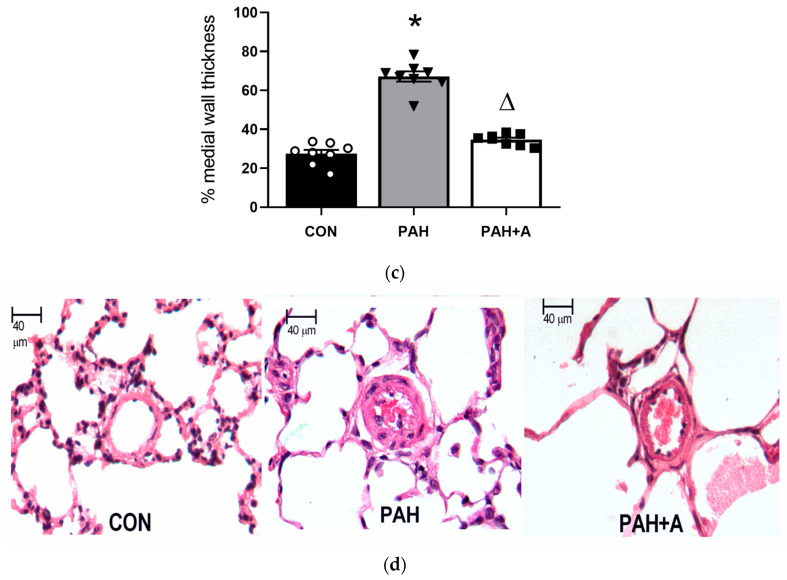
Validation of the establishment of PAH. (**a**) Survival percentage, (**b**) RV hypertrophy, (**c**) percentage of thickening of pulmonary arterial walls, and (**d**) representative images of the pulmonary arteries of the control (CON), with saline solution as the vehicle; PAH group (PAH) induced by 60 mg/kg/s.c. of MCT; and PAH with 16 mg/kg of allicin (PAH + A). Data are presented as mean ± standard error. Differences were tested by ordinary one-way ANOVA followed by the Bonferroni post hoc test for multiple comparisons, and significant differences between groups were set at *p* ≤ 0.05 n = 8, * *p* ≤ 0.05 vs. control; ^Δ^ *p* ≤ 0.05 vs. PAH.

The MCT-induced PAH model shares similarities with human PAH regarding hemodynamic and histopathological severity and high mortality. Our first result was at the macroscopic level and was related to survival (Figure 1a). In the control group, all the animals survived until the end of the protocol (Figure 1a). In contrast, the MCT-induced PAH model had 60% survival by the end of the follow-up. Surprisingly, allicin treatment halted the high mortality of MCT-induced PAH, and 90% of the animals survived by the end of the study (Figure 1a).

RV hypertrophy and the remodeling of the pulmonary arteries (arterial wall thickening) were evaluated to demonstrate the successful development of arterial hypertension. First, RV hypertrophy was evaluated using the Fulton index (RV/LV + S). We showed that MCT administration induced RV hypertrophy compared with the control group (0.55 ± 0.030 vs. 0.33 ± 0.01) (*p* < 0.05), while RV hypertrophy in the allicin-treated group (PAH + A) was prevented compared with the untreated group (0.39 ± 0.01 vs. 0.55 ± 0.030 (Figure 1b) (*p* < 0.05). Second, we evaluated the arterial wall thickening in lung tissue. We noted a narrower vascular lumen in the MCT-induced PAH group compared to the control group (67.18 ± 2.63 vs. 27.43 ± 1.47) (Figure 1c,d) (*p* < 0.05). Allicin treatment prevented the vessel wall thickening compared with the untreated MCT-induced PAH group (34.71 ± 1.02 vs. 67.18 ± 2.63) (Figure 1c,d) (*p* < 0.05). In summary, the results demonstrate that RV hypertrophy and pulmonary vascular remodeling, both parameters used as gold standards to validate the establishment of PAH in experimental models, were modified by allicin administration.

**Figure 2:**
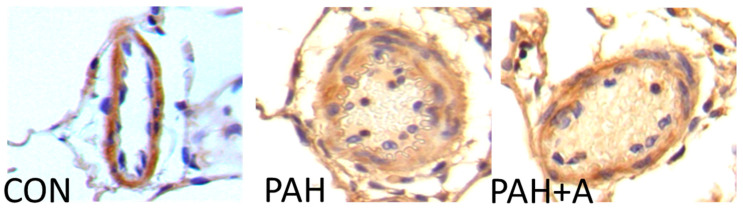
Representative micrographs of immunohistochemistry detection of the effects of allicin on the alpha-smooth muscle actin (α-SMA) in lung tissue. α-SMA detection clearly shows the hyperplastic muscular layer of the PAH group. Immunoblotting assay of control group (CON), with saline solution as the vehicle; PAH group (PAH) induced by 60 mg/kg/s.c; and PAH with 16 mg/kg of allicin (PAH + A). All micrographs 40×.

Some of the structural changes associated with vascular remodeling and vasoconstriction in PAH include smooth muscle cell proliferation and smooth muscle cell hypertrophy. In this context, we determined the alpha-smooth muscle actin expression as a marker of artery muscularization through immunohistochemistry. The results showed a stronger positivity to α-SMA in slices of PAH than in the control group, which was decreased with the allicin treatment (Figure 2).

### 2.2. Effect of Allicin on Renin–Angiotensin System Components at the Systemic and Pulmonary Level in Rats with PAH

To evaluate the RAS activation in PAH, we assessed the angiotensin II (Ang II) and angiotensin 1-7 (Ang-(1-7) concentrations in serum and lung tissue, as well as the expressions of angiotensin-converting enzyme 2 (ACE2), angiotensin II type 1 receptor (AT1R), and angiotensin II type 2 receptor (AT2R) in the lung.

**Figure 3:**
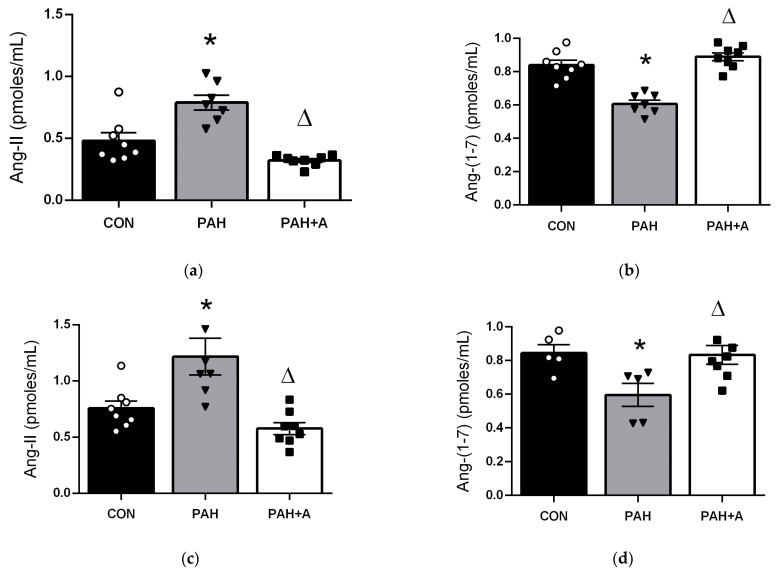
Allicin effects on angiotensin concentration at the systemic and local level. Ang-II concentration in (**a**) lung tissue and (**c**) serum. Ang-(1-7) concentration in (**b**) lung tissue and (**d**) serum. Control group (CON), with saline solution as the vehicle; PAH group (PAH) induced by 60 mg/kg/s.c. of MCT; and PAH with 16 mg/kg of allicin (PAH + A). Data are presented as mean ± standard error. Differences were tested by ordinary one-way ANOVA followed by the Bonferroni post hoc test for multiple comparisons, and significant differences between groups were set at *p* ≤ 0.05. n = 8, * *p* < 0.05 vs. control; ^Δ^ *p* < 0.05 vs. PAH.

We showed that PAH increased Ang-II concentration in lung tissue (0.78 ± 0.06 vs. 0.47 ± 0.06 pmoles/mL) and serum (1.21 ± 0.16 vs. 0.75 ± 0.064 pmoles/mL) compared with the control group (*p* < 0.05). In contrast, allicin treatment prevented the increase in Ang-II in lung tissue (0.32 ± 0.015 vs. 0.78 ± 0.06 pmoles/mL) and serum (0.57 ± 0.05 vs. 1.21 ± 0.16 pmoles/mL) (*p* < 0.05) (Figure 3a,c). In addition, in the PAH group, the concentration of Ang-(1-7) in lung tissue (0.60 ± 0.23 vs. 0.83 ± 0.029 pmoles/mL) and serum (0.59 ± 0.068 vs. 0.84 ± 0.49 pmoles/mL) decreased compared with the control group (*p* < 0.05). In contrast, allicin administration preserved the Ang-(1-7) concentration in the lung (0.88 ± 0.02 vs. 0.60 ± 0.23 pmoles/mL) and serum (0.83 ± 0.05 vs. 0.59 ± 0.068 pmoles/mL) of monocrotaline-induced PAH rats (Figure 3b,d).

**Figure 4:**
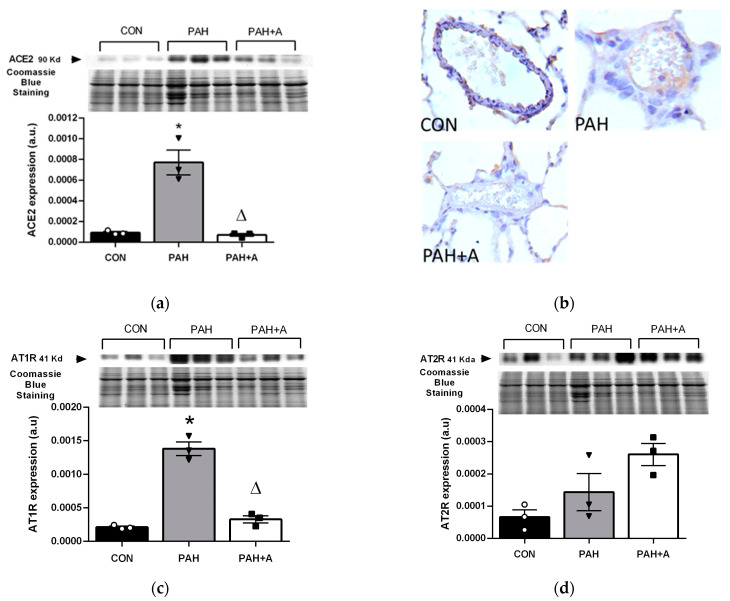
Effects of allicin on the protein expressions of ACE2, AT1R, and AT2R in lung tissue. Immunoblotting assay of (**a**) ACE2 and (**b**) representative micrographs of immunohistochemistry detection, and (**c**) protein expression of AT1R and (**d**) AT2R in lung tissue. Control group (CON), with saline solution as the vehicle; PAH group (PAH) induced by 60 mg/kg/s.c; and PAH with 16 mg/kg of allicin (PAH + A). For Western blotting, three randomly selected samples per group were analyzed. All micrographs 40×. Data are presented as mean ± standard error and were analyzed using the Kruskal–Wallis test. Significant differences between groups were set at *p* ≤ 0.05. n = 3, * *p* < 0.05 vs. CON; ^Δ^ *p* < 0.05 vs. PAH.

Subsequently, we determined the protein expression of ACE2 and the angiotensin II receptors (AT1R and AT2R) in the lung using an immunoblotting assay. In the PAH group, the protein expression of ACE2 in lung tissue was increased in comparison to the control group (*p* < 0.05) (Figure 4a). According to immunohistochemistry, activated cuboid endothelial cells showed stronger positivity (Figure 4b). In the PAH+A group, the ACE2 expression at the protein level determined by Western blot and immunohistochemistry was maintained at the same level as the control group, preventing the PAH-induced overexpression (Figure 4a,b) (*p* < 0.05). Similarly, the protein expression of AT1R in the PAH group was increased compared to the control group (*p* < 0.05) (Figure 4c), and allicin treatment prevented AT1R protein overexpression. In contrast, AT2R expression was not modified by PAH; neither allicin treatment produced a significant change, although an upward trend was observed (Figure 4d).

### 2.3. Effects of Allicin on Endothelial Function Markers in Serum and Lung Tissue of Rats with PAH

During the pathogenesis of PAH, the endothelial function plays a key role. Thus, we assessed NO, tetrahydrobiopterin (BH4), and dihydrobiopterin (BH2) as systemic and pulmonary markers of endothelial function.

**Figure 5:**
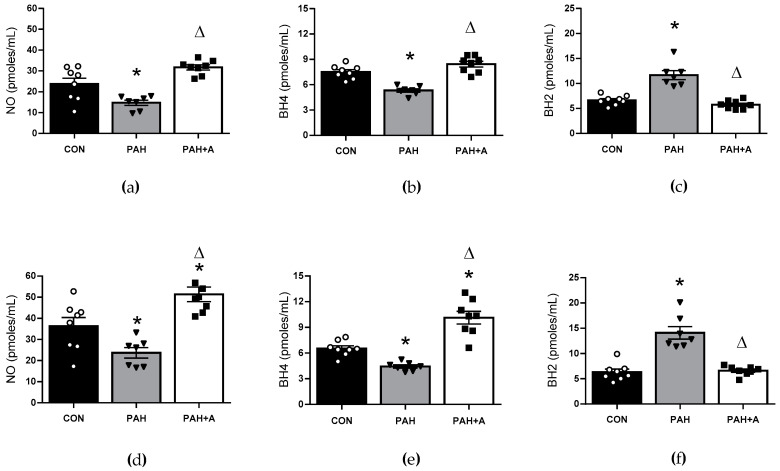
Allicin effects on endothelial function markers in lung and serum in PAH. Concentration of (**a**) NO, (**b**) BH4, and (**c**) BH2 in lung tissue. Concentrations of (**d**) NO, (**e**) BH4, and (**f**) BH2 in serum. Control group (CON), with saline solution as the vehicle; PAH group (PAH) induced by 60 mg/kg/s.c; and PAH with 16 mg/kg of allicin (PAH+A). Data are presented as mean ± standard error. Differences were tested by ordinary one-way ANOVA followed by the Bonferroni post hoc test for multiple comparisons, and significant differences between groups were set at *p* ≤ 0.05. n = 8, * *p* < 0.05 vs. control; ^Δ^ *p* < 0.05 vs. PAH.

We observed that NO concentration decreased in the lung tissue (14.71 ± 1.26 vs. 23.72 ± 2.82 pmoles/mL) and serum (23.66 ± 2.47 vs. 36.30 ± 4.04 pmoles/mL) of the PAH group. Treatment with allicin preserved NO in the lung (31.72 ± 1.22 vs. 14.71 ± 1.26 pmoles/mL), while in the serum (51.40 ±3.45 vs. 23.66 ± 2.47 pmoles/mL), allicin significantly increased NO concentration (*p* < 0.05) (Figure 5a,d). To analyze the functional status of endothelial nitric oxide synthase as a NO-releasing source, biopterins BH4 and BH2 were determined in lung tissue and serum. Again, BH4 concentration decreased in the PAH group (*p* < 0.05) compared with the control group in lung tissue (5.316 ± 0.20 vs. 7.50 ± 0.27 pmoles/mL) and serum (4.42 ± 0.19 vs. 6.53 ± 0.316 pmoles/mL). In contrast, allicin treatment preserved the concentration of BH4 in the lung (8.436 ± 0.33 vs. 5.316 ± 0.20 pmoles/mL) and significantly increased its concentration in the serum of PAH rats (10.14 ± 0.76 vs. 4.42 ± 0.19 pmoles/mL) (*p* < 0.05) (Figure 5b,e). As was expected, BH2 was increased in lung (11.66 ± 0.88 vs. 6.26 ± 0.34 pmoles/mL) and serum from the PAH group (14.08 ± 1.23 vs. 6.31 ± 0.60 pmoles/mL) (*p* < 0.05) when compared with the control group, and allicin maintained those concentrations similar to the control group levels in lung (5.75 ± 0.3 pmoles/mL) and serum (6.60 ± 0.32 pmoles/mL) (*p* < 0.05) (Figure 5c,f).

The results suggest that the allicin treatment prevented systemic and lung endothelial dysfunction during PAH by preserving NO bioavailability, likely mediated by maintaining the eNOS in the coupled state (increasing synthesis or reducing degradation).

In the NO-mediated endothelium-dependent vasodilation pathway, PDE5 and cGMP are closely related and play a fundamental role in the relaxation of vascular smooth muscle. Thus, we determined the protein expression of PDE5 in lung tissue by Western blot and immunohistochemistry and quantified the cGMP concentrations in the tissue and serum of rats with PAH.

**Figure 6:**
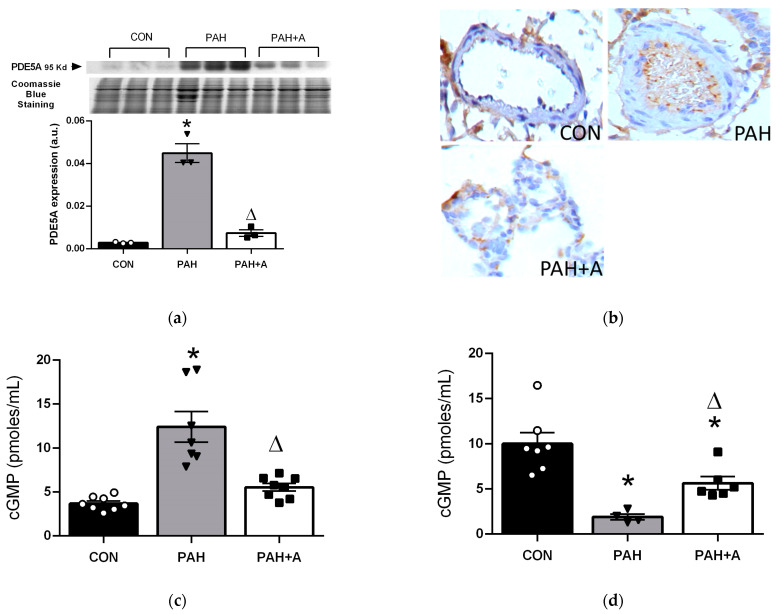
Allicin effects on PDE5 and cGMP. (**a**) PDE5 protein expression in lung tissue and (**b**) representative micrographs of immunohistochemistry detection. Concentrations of cGMP in (**c**) lung and (**d**) serum. Control group (CON), with saline solution as the vehicle; PAH group (PAH) induced by 60 mg/kg/s.c; and PAH with 16 mg/kg of allicin (PAH + A). For Western blotting, three randomly selected samples per group were analyzed. All micrographs 40x. Data are presented as mean ± standard error and were analyzed using the Kruskal–Wallis test. Significant differences between groups were set at *p* ≤ 0.05. n = 3, * *p* < 0.05 vs. CON; ^Δ^ *p* < 0.05 vs. PAH. Data of GMP are presented as mean ± standard error. Differences were tested by ordinary one-way ANOVA followed by the Bonferroni post hoc test for multiple comparisons, and significant differences between groups were set at *p* ≤ 0.05. n = 8, * *p* < 0.05 vs. CON; ^Δ^ *p* < 0.05 vs. PAH.

In the present study, we showed that PDE5 increased in the PAH group compared to the control group (*p* < 0.05). Allicin treatment preserved the low PDE5 levels in PAH + A group (*p* < 0.05) (Figure 6a). These results were in line with the immunohistochemistry analysis, which showed an increase in the positive cells to PDE-5 in the group of PAH compared with the control group and was prevented with the allicin treatment (Figure 6b). To further analyze the participation of PDE5, we determined its activity by measuring cGMP concentrations in lung tissue and serum. Lung cGMP concentrations in the PAH group were significantly higher compared to the control group (12.41 ± 1.72 vs. 3.7 ± 0.27 pmoles/mL) (*p* < 0.05), and allicin administration in PAH rats preserved cGMP concentrations in levels similar to the control group (5.54 ± 0.42 vs. 12.41 ± 1.72 pmoles/mL), being significantly lower compared to the PAH group (*p* < 0.05) (Figure 6c). In contrast, in the serum, we observed the opposite behavior. Thus, PAH induced a significant decrement in cGMP concentrations compared to the control group (1.90 ± 0.32 vs. 10.02 ± 1.23 pmoles/mL) (*p* < 0.05), while allicin treatment partially prevented the fall of cGMP concentrations (5.64 ± 0.73 vs. 1.90 ± 0.32 pmoles/mL) (*p* < 0.05) (Figure 6d).

### 2.4. Effects of Allicin on Oxidative Stress Markers in Lung Tissue and Serum of Rats with PAH

**Figure 7:**
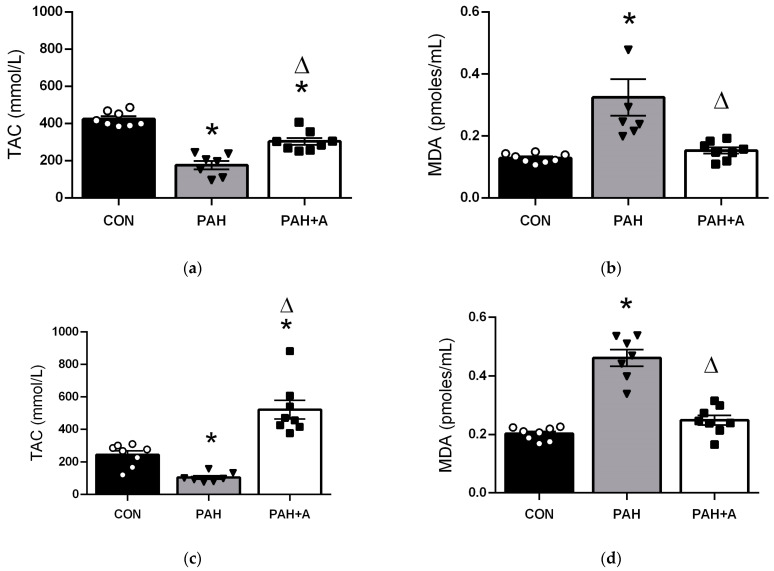
Effects of allicin in total antioxidant capacity (TAC) and malondialdehyde (MDA) as oxidative stress markers. The concentration of (**a**) TAC and (**b**) MDA) in the lung. The concentration of (**c**) TAC and (**d**) MDA in serum. Control group (CON), with saline solution as the vehicle; PAH group (PAH) induced by 60 mg/kg/s.c; and PAH with 16 mg/kg of allicin (PAH + A). Data are presented as mean ± standard error. Differences were tested by ordinary one-way ANOVA followed by the Bonferroni post hoc test for multiple comparisons, and significant differences between groups were set at *p* ≤ 0.05. n = 8, * *p* < 0.05 vs. control; ^Δ^ *p* < 0.05 vs. PAH.

It is well known that OS contributes to endothelial dysfunction and other deleterious effects of PAH, such as arterial wall remodeling. In addition, we observed that in the PAH group, there was increased RAS activity and endothelial dysfunction, both frequently related to OS. Thus, we explored allicin’s antioxidant effects by determining the total antioxidant capacity (TAC) and concentration of malondialdehyde (MDA), a marker of lipid peroxidation in serum and lung tissue. We showed a significant decrease in TAC in the lung of the PAH group compared to the control group (176.3 ± 22.51 vs. 423.9 ± 13.79 pmoles/mL) (*p* < 0.05). As an effective antioxidant, allicin partially preserved TAC in the lung (303.6 ± 18.82 vs. 176.3 ± 22.51 pmoles/mL) (Figure 7a) but further increased TAC in serum (Figure 6c) compared to PAH and the control group (521.3 ± 57.62 vs. 104.3 ± 11.16 pmoles/mL). These results were consistent with the increased concentration of MDA in the PAH group compared to the control group in lung tissue (0.3241 ± 0.028 vs. 0.12 ± 0.005 pmoles/mL) and serum (0.461 ± 0.28 vs. 0.20 ± 0.007 pmoles/mL). However, allicin treatment prevented the rise in MDA levels in lung tissue (0.15 ± 0.10 vs. 0.32 ± 0.05 pmoles/mL) and serum compared to the PAH group (0.24 ± 0.16 vs. 0.46 ± 0.28 pmoles/mL) (*p* < 0.05; Figure 7b,d).

### 2.5. Effect of Allicin on the Expression of Nrf2 and Keap1 Proteins in Lung Tissue of Rats with PAH

**Figure 8:**
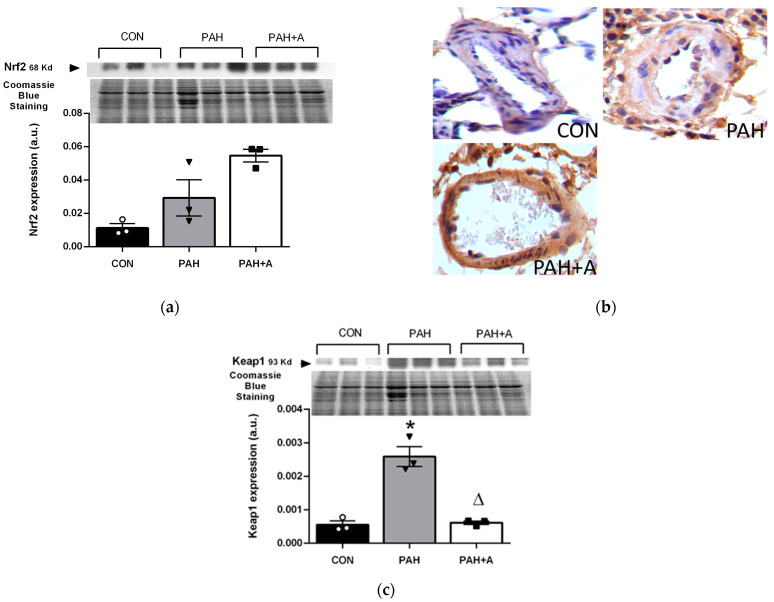
Effects of allicin on Nrf2 and Keap1 proteins on PAH. (**a**) Nrf2 by Western blot, and (**b**) representative micrograph of immunohistochemistry, where Nrf-2 is strongest in the arterial endothelium and muscle cells of PAH + A compared with the other groups. (**c**) Keap1 protein expression in lung tissue. Control group (CON), with saline solution as the vehicle; PAH group (PAH) induced by 60 mg/kg/s.c; and PAH with 16 mg/kg of allicin (PAH + A). For Western blotting, three randomly selected samples per group were analyzed. All micrographs 40×. Data are presented as mean ± standard error and were analyzed using the Kruskal–Wallis test. Significant differences between groups were set at *p* ≤ 0.05. n = 3, * *p* < 0.05 vs. control; ^Δ^ *p* < 0.05 vs. PAH.

The endogenous antioxidant system is regulated mainly by the Nrf2/Keap 1 system; therefore, we analyzed the expression of Nrf2 and Keap1 through immunoblotting and immunohistochemistry in lung tissue. The expression of Nrf2 in immunoblotting was not different among the groups (Figure 8a), although a tendency to increase was induced by allicin, which was most evident according to immunohistochemistry, which showed stronger immunostaining in the arterial endothelium and muscle cells (Figure 8b). In contrast, the expression of Keap1 in immunoblotting was significantly increased in the PAH group compared with the control group (*p* < 0.05). Allicin treatment prevented Keap1 overexpression induced by PAH (*p* < 0.05) (Figure 8b).

### 2.6. Effect of Allicin on the Expression of Hypoxia Markers in Serum and Lung Tissue of PAH

**Figure 9:**
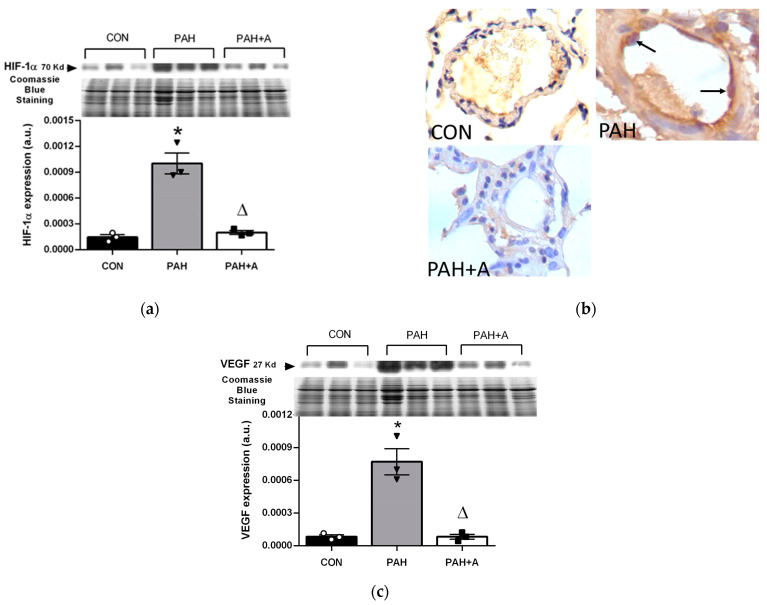
Allicin effects on markers of hypoxia in PAH. Protein expression of (**a**) hypoxia-inducible factor 1-alpha (HIF-1α) by Western blot, (**b**) representative micrograph of immunohistochemistry, where nuclear staining of HIF 1α is shown (black arrows) in PAH and was not observed in PAH + A group, and (**c**) protein expression of vascular endothelial growth factor (VEGF). Control group (CON), with saline solution as the vehicle; PAH group (PAH) induced by 60 mg/kg/i.p; and PAH with 16 mg/kg of allicin (PAH + A). For Western blotting, three randomly selected samples per group were analyzed. All micrographs 40×, except HIF-1 in PAH that is 100×. Data are presented as mean ± standard error and were analyzed using the Kruskal–Wallis test. Significant differences between groups were set at *p* ≤ 0.05. n = 3, * *p* < 0.05 vs. control; ^Δ^ *p* < 0.05 vs. PAH.

The vascular remodeling in PAH eventually leads to chronic hypoxia. In this context, the main cellular mechanisms of oxygen sensing involved in hypoxic response are the hypoxia-inducible factor 1alpha (HIF-1α) and the vascular endothelial growth factor (VEGF). Therefore, this study evaluated the protein expression of HIF-1α and VEGF in lung tissue. MCT-induced PAH increased the expression of HIF-1α and VEGF proteins compared with the control group (*p* < 0.05) (Figure 9a–c); endothelial and macrophages showed strong HIF-1α immunostaining of nucleus and cytoplasm. However, allicin treatment fully prevented the overexpression of HIF-1α and VEGF proteins compared with the PAH group (*p* < 0.05) (Figure 9a–c). The immunohistochemistry analysis shows an increase in the positive cells to HIF-1α in the group of PAH (endothelial and macrophages) compared to the control group, which was prevented with the allicin treatment (Figure 9b).

## 3. Discussion

Our main findings were that allicin administration through the modulation of several cellular mechanisms induced protective effects in an experimental model of PAH. First, we showed that oral administration of allicin encouraged body weight gain and survival by preventing the increase in pulmonary arterial medial wall thickness and RV hypertrophy. Subsequently, allicin prevented vasoconstriction through two mechanisms: (1) preserving low levels of Ang II concentration in serum and lung tissue and (2) stimulating vasodilation by preserving Ang-(1-7) and NO concentrations in serum and lung tissue. Further, allicin administration preserved serum cGMP concentration, while it decreased in lung tissue. The allicin administration preserved the protein expressions of ACE2, AT1R, and PDE5 at low levels. The reduction in oxidative stress markers in the lung and tissue demonstrated the antioxidant activity of allicin. Likewise, allicin prevented the overexpression of HIF-1α and VEGF, suggesting improved lung oxygenation.

PAH is a chronic, multifactorial, and fatal disease. The development of PAH could be due to unknown causes, genetic mutations, changes in gene expression (DNA, RNA), secondary to other diseases, or even drug intake [4,5,6,7,8]. Recent studies about methylation, deacetylation, and miRNA expression have demonstrated the role of epigenetic, transcriptional, and post-transcriptional mechanisms as critical factors involved in the development, maintenance, and progression of PAH. On the other hand, other pathogenic mechanisms associated with PAH development include endothelial dysfunction, oxidative stress (OS), vascular constriction, inflammation, and fibrosis [4,5,6,7,8].

Recent studies report the activation of the RAS in patients with PAH, as well as in experimental models in vivo and in vitro. In this context, it is well known that the activation of the RAS produces deleterious effects such as vasoconstriction, oxidative stress, inflammation, vascular remodeling, and cardiac hypertrophy [13,14]. Therefore, preventing the activation of the RAS could be a target to delay the progression of PAH. Previously, it was demonstrated that allicin exerts therapeutic effects by reducing hypertension, attenuating vascular reactivity to Ang II and cardiac hypertrophy, and modulation of Ang II receptor expression [22]. Also, an in silico analysis showed that allicin inhibits the action of AT1R via a similar mechanism to losartan [22,23]. Thus, we assessed whether the components of the RAS are present in the experimental PAH and tested whether allicin through modulation of the RAS induces beneficial effects. The histopathology analysis showed a thickening of the medial wall of the pulmonary arteries, which induced RV hypertrophy. RHF was likely the cause of the 40% death rate observed in our experimental group of MCT [30]. These results agreed with the characteristic phenotype of PAH [3,30,31,32,33].

Further, increased Ang II concentrations in serum and lung tissue were related to increased AT1R expression in the lung. These effects were prevented with allicin. It is well known that Ang II mediates its pathological effects by the interacting with its receptors. In this context, recent studies provide evidence that the modulation of RAS components induces beneficial pulmonary outcomes with no adverse effects on systemic blood pressure [33,34].

In the vasculature, there is a balance between vasodilators and vasoconstrictors, so vascular smooth muscle vasoconstriction arises in response to Ang II and vasodilates in response to NO. Both molecules have antagonistic effects on vascular tone and vascular remodeling [35]. Our results showed a decrement in NO concentrations in the serum and tissue of PAH rats. These effects were probably due to an uncoupled activity of eNOS because the BH4 concentrations were decreased, while BH2 increased in lung and serum from experimental PAH.

Additionally, our results showed an increase in PDE5 expression, suggesting that the vasodilation signaling cascade also is altered downstream in lung tissue. PDE5 breaks down cGMP, thus producing smooth muscle contraction and vasoconstriction [3]. In the present study, despite the increase in PDE5, cGMP concentrations in lung tissue were increased, whereas those in the serum were decreased. This apparent conflict could be explained by the fact that cGMP can also be produced from the particulate guanylyl cyclase (pGC), which is part of the natriuretic peptide receptors A and B that can be activated via atrial and brain natriuretic peptides (ANPs and BNPs) respectively [36]. In this regard, pro-BNP levels are highly prognostic of PH progression and indicate patients at the highest risk [37].

Allicin improves vasodilation in lung tissue and at the systemic level by preventing increased Ang II concentrations and AT1R overexpression. Moreover, the decrement of the vasodilative substance Ang-(1-7) in PAH was prevented with allicin. Additionally, the BH4 and NO concentrations were increased with allicin, while BH2 concentrations were decreased, suggesting an improvement in eNOS activity. The vasodilative effects of allicin also were supported by the increase in cGMP in the lung. We hypothesize that the allicin treatment improved endothelial function since the evaluated markers suggest increased NO bioavailability, likely mediated by maintaining coupled eNOS (increasing synthesis or reducing degradation).

On the other hand, vascular remodeling causes alterations not only at the arterial pressure level but also causes endothelial dysfunction, infiltration of inflammatory cells, inflammation, fibrosis, OS, and hypoxia, which feedback and contribute to the progression of the disease. Chronic activation of these stimuli gradually results in pulmonary artery lumen occlusion, increasing pulmonary vascular resistance (PVR) and PAH. Accordingly, patients with PAH reported increased markers of OS in urine, plasma, and lung tissue [38,39].

Numerous pieces of evidence have shown that Ang-II-induced ROS formation leads to cardiovascular diseases, including cardiac hypertrophy and heart failure [40,41,42]. Ang II plays a key role in hypertension and atherosclerosis in animal models and humans, due to the interaction with the AT1 receptor, which increases ROS production in the vessel wall by activating NADH/NADPH oxidase [43,44].

The experimental model of PAH showed an increase in systemic and tissue OS markers (TAC and lipid peroxidation), which was associated with the increase in Keap1, the protein repressor of Nrf2 factor, and the master regulator of transcription of endogenous antioxidant enzymes (catalase, superoxide dismutase, glutathione peroxidase, etc.) [2]. Allicin prevented OS by increasing TAC and inhibiting lipid peroxidation, RAS, and Keap1, thus increasing the synthesis of antioxidant enzymes, and likely through directly neutralizing free radicals and ROS [2,23,29,45]. Other studies reported that allicin exerts beneficial effects in experimental PAH and CKD through the prevention of cardiac hypertrophy; improvement of endothelial, cardiac, and renal function; blocking of the interaction of Ang II with the AT1 receptor; and the modulation of oxidative status by the Nrf2/Keap1 pathway [22,23,25,46].

It has been reported that inflammation increases cellular metabolism, leading to decreased local oxygen bioavailability, creating hypoxic conditions and OS [47,48]. HIF-1α is a hypoxia response factor that regulates various cellular processes, including cell proliferation, inflammation, and fibrosis, which play a crucial role in vascular remodeling [48]. One target gene of HIF-1α is VEGF, which promotes angiogenesis to increase oxygen delivery to hypoxic tissue [48]. Our results showed increased HIF-1α and VEGF in lung tissue from the PAH experimental model, which can be a response to hypoxia in the lung induced by vascular remodeling, inflammation, and OS. In lung tissues and isolated pulmonary arterial endothelial cells from patients with idiopathic PAH and experimental models, the expression of HIF-2α was found to increase [31], suggesting that hypoxic factors are involved in PAH. On the other hand, activating the RAS in PAH also leads to the activation of HIF-1α [49]. It has been reported that Ang II and several cytokines induce HIF-1α, even in the absence of hypoxia, thus leading to further vascular remodeling, inflammation, and OS injury [50,51].

Allicin treatment prevented the increase in HIF-1α and VEGF, possibly mediated by its effects on inflammation, OS, and RAS. Also, our results suggest that the reduction in HIF-1α possibly is involved in the reduction in the thickening and remodeling of the pulmonary arteries, thus improving tissue oxygenation, reducing RV hypertrophy, and increasing survival in PAH. It was reported that the pharmacological inhibition of HIF-2α in PAH reduces hypertrophy, right ventricular systolic pressure, fibrosis, and pulmonary vascular remodeling; prevents RHF; and favors survival [31]. Also, it has been described that hypoxia-induced PAH is a stimulus for the appearance of smooth muscle-like cells in vessels. In this context, we observed an increase in the pulmonary arterial medial wall thickness and an increase in α-SMA in the group of PAH. α-SMA is a marker of endothelial-to-mesenchymal transition and a marker of smooth muscle cells, suggesting the muscularization of arteries in the lung. Such an effect is associated with HIF-1α expression and the occlusion of the vascular lumen in PAH [52,53]. The allicin treatment decreased the expression of HIF-1α and α-SMA, which was related to the reduced thickening of the arterial wall.

Although PAH has several causal factors, the results are pulmonary vascular remodeling and RV hypertrophy. Thus, increased right ventricular afterload may cause progressive maladaptation, and RHF is the expected outcome [3]. The deterioration of the vasodilator and antioxidant systems and the activation of proinflammatory and profibrotic signaling pathways contribute significantly to the progression and outcome of the disease. So far, therapeutic strategies for treating PAH are mainly focused on improving pulmonary vasculature vasodilation mediated by the NO pathway; endothelin, prostaglandin, thromboxane receptors; and PDE5 inhibitors, and more than one drug is needed to correct them [54]. Recent studies suggest that the therapy for PAH should also improve vasodilation and stop the remodeling of the pulmonary arteries, even though it has been proposed that the correct approach must be focused on both targets to enhance therapeutic efficacy [54].

Although current treatments have helped to improve the quality of life of the patient with PAH, these are still not entirely effective since it is known that the response to treatments is variable, sometimes showing little or no effectiveness, and can induce side effects [8]. Therefore, considering the complexity and multicausality of PAH, it is imperative to find therapeutic options for improving the outcomes that help to control or even cure this devastating disease adequately.

Our results and the various scientific reports support the concept that allicin represents a potential therapeutic option for treating PAH, since it promotes vasodilatation mediated by the NO pathway, modulates RAS, regulates the oxidant status, and improves lung oxygenation. These effects are essential to maintaining endothelial function and controlling vascular remodeling. Additionally, allicin decreases pulmonary vascular remodeling, fibrosis, inflammation, and RV hypertrophy [6], which together will result in a better prognosis and life expectancy, decreasing the use of polypharmacy.

Our study has several limitations. Allicin treatment was preventive rather than curative, so future studies should be conducted to evaluate allicin’s effect on established PAH. A control to assess the effects of allicin on a healthy group was not included, but other studies reported that allicin did not induce changes in systolic blood pressure, heart rate in gross and whole hearts, or hypertrophic markers compared with the untreated control group [55]. Pulmonary hemodynamic parameters (RVSP, PVR, or PAP) as indicators of PAH were not measured; instead, we used the Fulton index (RV/LV + S), a well-known marker of RV hypertrophy which correlates with alterations in pulmonary hemodynamics in experimental PAH [56,57,58]. This index was increased in the MCT group when compared with the control group. Also, the monocrotaline-induced PAH model develops hemodynamic alterations in the right ventricle, such as increased mean pressure, peak pressure, end-stroke volume, end-diastolic volume, stroke work, and stroke volume [32,59]. These alterations are associated with a greater thickness of the medial wall of the pulmonary artery and RV hypertrophy [32,59]. Together, these parameters are indicators of PAH. Therefore, our study considered RV hypertrophy and arteriolar medial wall thickness indicators of PAH. Despite the limitations of our study, we observed that PAH was successfully established and the allicin treatment was beneficial, reflected in better survival, which is also an indicator of improved health.

## 4. Materials and Methods

### 4.1. Experimental Model of PAH and Groups

**Figure 10:**
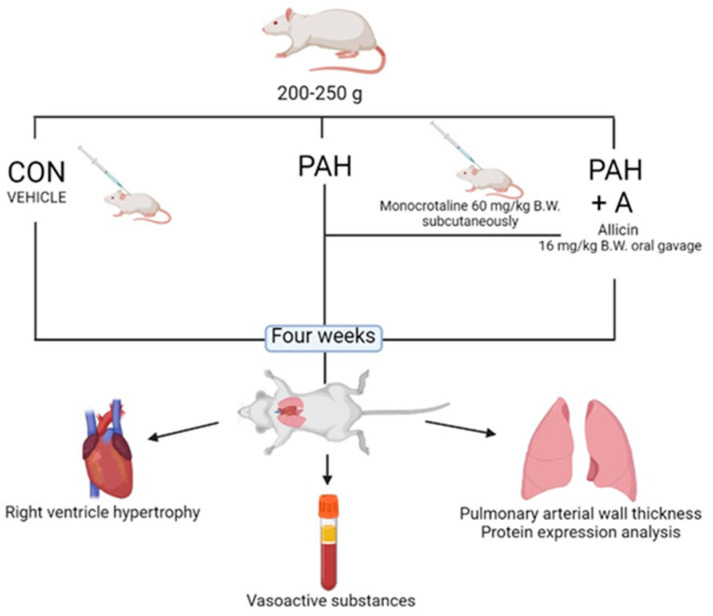
Experimental design.

Male Wistar rats weighing 200–250 g were randomly divided into three groups (n = 8 per group): control (CON) (saline solution), pulmonary arterial hypertension (PAH) (a single subcutaneous injection of monocrotaline (60 mg/kg body weight), catalog C2401-1G Sigma-Aldrich, St. Louis, MO, USA), and PAH plus allicin administration (16 mg/kg/oral gavage) (PAH + A) from the beginning of the study (Figure 10). The experimental groups were maintained with commercial pellets for rodents (PMI Nutrition International, Inc., LabDiet 5008, Richmond, IN, USA) and water ad libitum, under 12 h light/dark cycles and room temperature of 22 °C for four weeks.

This research protocol was reviewed and approved by the Internal Animal Care and Use Committee of Instituto Nacional de Cardiología Ignacio Chavez (INC/CICUAL/001/2021) and conducted following the Mexican Official Norm (NOM-062-ZOO-1999) for the production, care, and use of laboratory animals and the Guide of Care and Use of Laboratory Animals 8th Edition.

### 4.2. Allicin

Synthetic allicin (purity of 90–92%) was produced by the oxidation of diallyl disulfide (DADS), as previously reported [23]. Allicin was resuspended in water at 1.5% (*w*/*v*) for stabilization and storage and kept in 1 mL aliquots at −70 °C until used.

### 4.3. Assessment of RV Hypertrophy

For the hypertrophy analysis of RV, the rats were anesthetized with 50 mg/kg of ketamine and 10 mg/kg of xylazine intraperitoneally. The lack of pain response was assessed using the pedal withdrawal reflex. A sample of blood (using K-EDTA as an anticoagulant) was obtained by cardiac puncture after mid-thoracotomy. The heart and lungs were dissected and washed with a 0.9% saline solution to remove the excess blood. Later, the right ventricle was separated from the left ventricle plus septum (LV + septum). The Fulton index (FI; the ratio of RV to LV + septum weights) was used as an RV hypertrophy indicator [56]. Finally, the tissues were immediately frozen and kept at −70 °C until analysis.

### 4.4. Measurement of Pulmonary Arterial Medial Wall Thickness and Immunohistochemistry Assays

Pulmonary circulation was flushed with 5 mL buffered saline. The right and left lungs were immediately separated and frozen in liquid nitrogen for further measurements and histological analysis. For morphometric analysis, the left lung was fixed in 4% neutral buffered formalin, processed for histology, and stained with hematoxylin and eosin according to standard procedures. Sixty to eighty intra-acinar arteries/rat were analyzed to calculate the medial wall thickness. Intra-acinar arteries with an external diameter of less than 50 μm were included and microphotographed with 40× magnification. An automated histology system (Leica Microsystem Imaging Solutions LTD, Cambridge, UK) was used for the morphometric analysis. The wall thickness was calculated using the following formula: [(2 × medial wall thickness/external diameter) × 100)] and expressed as a percentage as previously reported [60].

Lung sections of 4 µm were mounted on silane-covered slides for the immunohistochemistry assay. After deparaffination and rehydration, heat-induced antigen retrieval was performed with ImmunoDNA Retriever Citrate (Bio SB, Santa Barbara, CA, USA). Then, peroxidase activity was blocked by incubation with 0.3% H_2_O_2_ and blocked with Background Sniper (Biocare Medical, Pacheco, CA, USA). Lung sections were incubated overnight at room temperature with rabbit polyclonal IgG antibody against alpha-smooth muscle actin (sc-130619, Santa Cruz Biotechnology, Dallas, TX, USA) and Nrf2 (SAB5700720, Merck, St. Louis, MO, USA) and mouse polyclonal IgG antibody against ACE-2 (sc-390851, Santa Cruz Biotechnology), PDE5A (sc-398747, Santa Cruz Biotechnology), and HIF-1α (sc-13515, Santa Cruz Biotechnology) diluted 1:100. This was followed by incubation with mouse/rabbit Polydetector DAB HRP (Bio SB). Bound antibodies were detected with diamino-benzidine (Merck) and counterstained with hematoxylin.

### 4.5. Concentrations of Nitric Oxide (NO), Tetrahydrobiopterin (BH4), Dihydrobiopterin (BH2), Cyclic Guanosine Monophosphate (cGMP), and Malondialdehyde (MDA) and Total Antioxidant Capacity (TAC)

In serum or lung homogenates, 100 µL of a vanadium chloride (III) solution at 0.8% (*w*/*v*) in 1M phosphoric acid was added to 20 μL of the sample and homogenized. After this procedure, 50 μL of the 2% sulfanilamide solution in 5% phosphoric acid was added and vigorously mixed, and 50 μL of the 0.2% N-(1-naphthyl)-ethylenediamine solution in distilled water was added, gently homogenized, and allowed to stand for 45 min at room temperature, protected from light. After the incubation, 1 mL of deionized water was added, and the samples were read spectrophotometrically at 572 nm and 587 nm. NO, BH4, BH2, cGMP, MDA, and CAT concentrations were obtained indirectly by preparing a moisture-free HPLC-grade sodium nitrite standard curve ranging from 0 to 200 pmol/mL. The absorbance difference (572–587 nm) was considered for the calculations [61].

### 4.6. Determination of Angiotensin II and 1-7 (Ang II and Ang 1-7)

Ang II and Ang–(1-7) were simultaneously determined in the rat serum or lung homogenate (50 μL) by capillary zone electrophoresis with UV detection by the photodiode array. Samples were deproteinized with cold methanol and 20% trichloroacetic acid in a 10:1 ratio, then were centrifuged at 16,000× *g* for 15 min at 10 °C and filtered with 0.22 µm nitrocellulose membrane filters, diluted 1:10 with cold 0.1 M sodium hydroxide. The samples were passed through Cold Sep-Pak Classic C-18 cartridges and tested directly on the P/ACE^TM^ MDQ de Beckman Coulter system for previous capillary precondition by passing 1.0 M sodium hydroxide solution for 30 min, then deionized water for 30 min, and finally the running buffer (100 mM boric acid + 3 mM tartaric acid + 10 fM gold III chloride at pH 9.8) for 30 min. Samples were injected under hydrodynamic pressure at 0.5 psi/10 s. Separation was performed at 30 kV for 10 min at 200 nm at 20 °C. The capillary was washed between runs with 1.0 M sodium hydroxide for 2 min, deionized water for 2 min, and running buffer for 4 min. The results were expressed in pmoles/mL. Ang II and Ang 1-7 concentrations were determined using a standard curve [62].

### 4.7. Protein Expression Analysis

Lungs (3 randomly selected samples per group) were homogenized in lysis buffer (10 mM HEPES, 0.2% Triton X-100, 50 mM NaCl, 0.5 mM sucrose, 0.1 mM EDTA, protease, and phosphatase inhibitors) and later centrifuged at 10,000 rpm for 10 min at 4 °C. The supernatant was separated, aliquoted, and stored at −70 °C for further analysis. The Bradford method was used to determine total protein concentration. For each sample, 7.5 µg of total proteins was resolved by SDS-PAGE and then electro-transferred to a polyvinylidene fluoride membrane (Millipore Corp., Bedford, MA, USA). The antibodies purchased from Santa Cruz Biotechnology (Santa Cruz Biotechnology, Inc. CA, USA) were phosphodiesterase-5 (PDE5A): 398747 (1:5000) and angiotensin-converting enzyme 2 (ACE2): 390851 (1:5000). The antibodies purchased from Abcam (Cambridge, MA, USA) were angiotensin II type 1 receptor (AT1R) antibody: ab124734 (1:10,000) and angiotensin II type 2 receptor antibody (AT2R): ab92445 (1:10,000). The antibodies purchased from GeneTex (Irvine, CA, USA) were hypoxia-inducible factor 1-alpha (HIF-1α): GTX127309 (1:5000), Kelch-like ECH-associated protein 1 (KEAP1): GTX60660 (1:5000), nuclear factor erythroid 2-related factor 2 (Nrf2): GTX103322 (1:5000), and vascular endothelial growth factor (VEGF): GTX21316 (1:10,000). A horseradish peroxidase-conjugated secondary antibody and enhanced chemiluminescence reagents (Clarity Western ECL Substrate, Bio-Rad) were used for the detection of primary antibodies. The bands of interest were quantified using a Kodak Electrophoresis Documentation and Analysis System 290 (EDAS 290). As loading control, the total protein was determined by Coomassie Blue R-250 (Bio-Rad, Hercules, CA, USA) staining. The protein expression was expressed as the ratio of the protein interest sample to loading control in arbitrary units (a.u.).

### 4.8. Statistical Analysis

Results were expressed as the mean ± standard error and analyzed by one-way variance analysis (ANOVA), followed by the Bonferroni post hoc test. For protein expression analysis, the results were presented as mean ± standard deviation (S.D.) of three randomly selected samples per group. The non-parametric Kruskal–Wallis test analyzed the effects of allicin treatment on protein expression. The statistical analyses were performed using GraphPad Prism (San Diego, CA, USA) version 8.0. Values of *p* < 0.05 were considered statistically significant.

## 5. Conclusions

Allicin attenuated the vascular remodeling and RV hypertrophy in PAH through its effects on NO-dependent vasodilation, modulation of RAS, and amelioration of OS. Also, these effects could be associated with the modulation of HIF-1α and improved lung oxygenation. The global effects of allicin contribute to preventing endothelial dysfunction, remodeling of the pulmonary arteries, and RV hypertrophy, preventing heart failure, thus favoring survival. Although human studies are needed, the data suggest that, alone or in combination therapy, allicin may be an alternative in treating PAH if we consider that, similarly to current treatments, it improves lung vasodilation and increases survival. The use of allicin may be considered an option when there is a lack of efficacy, and where drug intolerance is observed, to enhance the efficacy of drugs, or when more than one pathogenic mechanism must be addressed.

## Data Availability

The data presented in this study are available in the article.
